# Extensively Current Activity of Transposable Elements in Natural Rice Accessions Revealed by Singleton Insertions

**DOI:** 10.3389/fpls.2021.745526

**Published:** 2021-09-28

**Authors:** Zhen Liu, Han Zhao, Yan Yan, Ming-Xiao Wei, Yun-Chao Zheng, Er-Kui Yue, Mohammad Shah Alam, Kwesi Odel Smartt, Ming-Hua Duan, Jian-Hong Xu

**Affiliations:** ^1^Hainan Institute, Zhejiang University, Sanya, China; ^2^Zhejiang Key Laboratory of Crop Germplasm, Institute of Crop Science, Zhejiang University, Hangzhou, China; ^3^Jiangsu Provincial Key Laboratory of Agrobiology, Institute of Biotechnology, Jiangsu Academy of Agricultural Sciences, Nanjing, China; ^4^Zhejiang Zhengjingyuan Pharmacy Chain Co., Ltd., Hangzhou, China; ^5^Hangzhou Zhengcaiyuan Pharmaceutical Co., Ltd., Hangzhou, China

**Keywords:** transposable element (TE), transposition activity, singleton insertion, unique insertion site, rice (*Oryza sativa*)

## Abstract

Active transposable elements (TEs) have drawn more attention as they continue to create new insertions and contribute to genetic diversity of the genome. However, only a few have been discovered in rice up to now, and their activities are mostly induced by artificial treatments (e.g., tissue culture, hybridization etc.) rather than under normal growth conditions. To systematically survey the current activity of TEs in natural rice accessions and identify rice accessions carrying highly active TEs, the transposon insertion polymorphisms (TIPs) profile was used to identify singleton insertions, which were unique to a single accession and represented the new insertion of TEs in the genome. As a result, 10,924 high-confidence singletons from 251 TE families were obtained, covering all investigated TE types. The number of singletons varied substantially among different superfamilies/families, perhaps reflecting distinct current activity. Particularly, eight TE families maintained potentially higher activity in 3,000 natural rice accessions. Sixty percent of rice accessions were detected to contain singletons, indicating the extensive activity of TEs in natural rice accessions. Thirty-five TE families exhibited potentially high activity in at least one rice accession, and the majority of them showed variable activity among different rice groups/subgroups. These naturally active TEs would be ideal candidates for elucidating the molecular mechanisms underlying the transposition and activation of TEs, as well as investigating the interactions between TEs and the host genome.

## Introduction

Transposable elements (TEs) or transposons are mobile DNA sequences that can jump from one location to another within the genome. Since the discovery of the first transposon in maize more than 70 years ago (McClintock, 1950), TEs have been shown to be ubiquitous in almost all eukaryotic species and frequently constitute large fractions of plant and animal genomes (Huang et al., 2012; Vitte et al., 2014). TEs are the major contributor to the variation in genome size between related species. The burst of three LTR-retrotransposon families results in massive genome expansion, which made the genome size of Australia wild rice (*Oryza australiensis*) twofold larger than that of Asian cultivated rice (*Oryza sativa*) (Piegu et al., 2006). As an important source of genetic variations, TEs have also been well documented to contribute to gene and genome evolution in a variety of ways, including insertion mutagenesis, chromosome rearrangement, and epigenetic regulation (Lisch, 2013; Bennetzen and Wang, 2014; Chuong et al., 2017; Song and Cao, 2017). Therefore, the study of TEs would give us a better understanding of genome evolution and genetic diversity.

Transposable elements can be divided into two classes according to their DNA or RNA intermediates (Wicker et al., 2007). Class I element or retrotransposon is mediated by RNA intermediate during reverse transcription process *via* the “copy and paste” mode. Class II element or DNA transposon is directly excised from the original position and integrates into new genomic location *via* the “cut and paste” mode. Both TE classes contain autonomous and non-autonomous elements. The autonomous one has the ability to encode various enzymes required for (retro)transposition, which is responsible for the movement of themselves and the corresponding non-autonomous counterparts.

To date, a large number of TEs have been identified from eukaryotic genomes, but most of them have already lost mobility, because TEs accumulated lots of mutations or were partially deleted by combinations (Ma et al., 2004). On the other hand, the host genomes have evolved multiple defense mechanisms including DNA methylation, chromatin modification, and RNA interference to efficiently silence TE activity to avoid the adverse consequences (Lisch, 2009; Cui et al., 2013; Cui and Cao, 2014; Fultz et al., 2015). Nevertheless, a few TEs are still active, such as *Ac*/*Ds* and *Spm*/*dSpm* in maize (McClintock, 1950; Wessler, 1988), *Tto1* and *Tnt1* in tobacco (Grandbastien et al., 1989; Hirochika, 1993), *P* elements in *Drosophila* (Rubin et al., 1982), *Alu*, LINE-1 and SVA (SINE/VNTR/*Alu*) in humans (Mills et al., 2007). These active TEs have drawn more attention as they continue to create new insertions in the genome to drive intraspecies diversity. They not only cause deleterious phenotypes or diseases by altering gene expression and function when integrated into genes (Iskow et al., 2010; Lee et al., 2012), but also produce potentially beneficial variants and participate actively in adaptive evolution (Gonzalez et al., 2008; Schrader and Schmitz, 2019). In addition, these active TEs have served as valuable resources to elucidate the molecular mechanisms underlying the transposition and activation of TEs.

Likewise, transposition activity has always been an active field in rice genomics research. However, due to the scarcity of active TEs and the technical difficulty in their identification, only a dozen active TE families have been discovered up to now. *Tos17*, belonging to the *Copia* superfamily, is the first active retrotransposon isolated from rice (Hirochika et al., 1996), which exhibits high level of transposition activity under tissue culture and has been used to induce rice mutants for functional genomics studies (Hirochika, 2001). Subsequently, the first active DNA transposon, *mPing* was discovered (Jiang et al., 2003). It is a non-autonomous tourist-like MITE (miniature inverted-repeat transposable element), derived from the perfect deletion of internal sequences of autonomous partner *Ping*. Its active mobilization has been demonstrated in three distinct situations: prolonged cell culture, anther-derived calli, and seeds exposed to γ-irradiation (Jiang et al., 2003; Kikuchi et al., 2003; Nakazaki et al., 2003). In the past decade, several other active TEs have been identified through characterization of spontaneous mutations or analysis of transcription profiles. These include *Copia* retrotransposons *Lullaby* (Picault et al., 2009), *Osr4*, *Osr13* (Cheng et al., 2015), *Osr7*, *Osr23* (Wang et al., 2009), *Gypsy* retrotransposons *Dasheng* (Cheng et al., 2015), LINE elements *Karma* (Komatsu et al., 2003), *LINE1-6_OS* (Cheng et al., 2015), and MITEs *dTok* (Moon et al., 2006), *nDart* (Tsugane et al., 2006), *nDaiZ* (Huang et al., 2009), *mGing* (Dong et al., 2012), *mJing* (Tang et al., 2019).

The emergence of new insertion in the genome is the most powerful and direct evidence to prove active transposition. The availability of large amount of resequenced data makes it possible to identify new insertions from numerous varieties from different species (Quadrana et al., 2016; Carpentier et al., 2019; Fuentes et al., 2019; Macko-Podgorni et al., 2019; Dominguez et al., 2020; Liu et al., 2020a, b). To fully understand the current activity of TEs in natural rice accessions and identify rice accessions carrying highly active TEs, we analyzed the comprehensive transposon insertion polymorphisms (TIPs) profile of 60,743 TE loci genotyped in 3,000 diverse accessions across five varietal groups [indica, japonica, aus/boro, basmati/sadri (aromatic) and intermediate type (admix)] (Liu et al., 2020b) to detect singleton insertions, which were unique to a single accession of the whole population and represented the new insertion of TEs in the genome. It was found that eight families maintained potential high activity in 3,000 natural rice accessions, and nine accessions contributed more than 50 singletons. These naturally active TEs would be ideal candidates for elucidating the molecular mechanisms underlying the transposition and activation of TEs, and help us fully understand the dynamics of rice TEs and their contribution to genetic diversity.

## Materials and Methods

### Screening and Filtering of Singleton Insertions

The comprehensive TIPs profile of 60,743 TE loci genotyped in 3,000 diverse accessions (including 1,760 indica, 843 japonica, 215 aus/boro, 68 basmati/sadri and 134 intermediate type accessions) was obtained by analyzing the resequencing data based on our previously developed pipeline^1^ (Liu et al., 2020b). A series of custom Perl scripts^2^ were written to check the presence/absence status of each insertion in 3,000 rice accessions, and we obtained the initial list of singleton insertions. Subsequently, these singletons were further filtered to eliminate low-quality and redundant singletons in the following aspects (Supplementary Figure 1). Firstly, considering that all resequenced rice samples were from homozygous accessions, TE insertions detected as heterozygosity were considered to be ambiguous and be discarded. Secondly, for a particular singleton, the high proportion of missing calls across the population would increase the likelihood that the insertion was not a singleton. Therefore, if one singleton had missing calls in more than 10% of rice accessions, it would be excluded from our dataset. Thirdly, if one singleton had no sufficient supporting reads (≥3 reads), it was likely to possess potentially higher prediction error rates and would be removed. Besides, if one singleton had two records corresponding to the forward and reverse direction, only one record of them was retained in the subsequent analysis to avoid double counting. In this way, we obtained a filtered dataset consisting of high-confidence non-redundant TE insertions.

### Integration Preference Analysis

To visualize the distribution of singletons from individual TE family in the genome, they were mapped to the 12 rice chromosomes *via* custom JavaScript scripts. Meanwhile, Perl scripts were written to calculate their density along the chromosome. Here, the genome was scanned in a 1 Mb sliding window with a 0.5 Mb increment. To compare the density between the chromosomal arm and pericentromere region, the annotation of the pericentromeric region in the pseudomolecules assembly was obtained from Rice Genome Annotation Project^3^. Then, the average values of all sliding windows located in the corresponding region were calculated. Finally, the paired *t*-test was used to test the significance of difference between two regions in the 12 rice chromosomes, and was carried out by the function *t* test in R (version 3.5.1).

### Enrichment Analysis for Rice Accessions Carrying Potentially Highly-Active Transposable Elements

To test whether the rice accession carrying potentially highly active (PHA) TEs were overrepresented in a particular varietal group, we preformed enrichment analysis for the TE family showing high activity in multiple rice accessions. The odds ratio (OR), a parameter used to measure the degree of enrichment, was calculated by the following equation: OR = [A/(B–A)]/[C/(3000–C)], where A represented the number of rice accession carrying the corresponding PHA TEs in a given varietal group, while B represented the total number of rice accession carrying the corresponding PHA TEs in 3,000 natural rice accessions; C represented the number of rice accessions owned by the variety group in 3,000 natural rice accessions. Then, the Fisher’s exact test was used to test the significance of the degree of enrichment, and was implemented by the function fisher test in R (version 3.5.1).

### Detection of Target Site Duplications

In our dataset, about half of singleton loci were identified simultaneously from both the forward and reverse directions around the TE insertion. Moreover, all of them belong to the non-reference insertion, which was present in the resequenced genome but absent from the reference genome. Therefore, we could detect the TSD of these singletons by comparing the sequences of the two genotypes with or without TE insertion. Briefly, when the two flanking sequences from both ends of a non-reference insertion are mapped to the reference genome, an overlapping region consisting of several bases usually appears as shown in Supplementary Figure 2, which corresponds to the TSD of the non-reference insertion. Based on this idea, we calculated the overlapping region for each singleton according to their coordinates on the genome detected from the forward and reverse direction. Then, the corresponding sequences were extracted from the reference genome. To understand their conservation, the WebLogo 3 (Schneider and Stephens, 1990; Crooks et al., 2004) was used to generate the sequence logo for the TSDs of individual TE family.

## Results

### Abundant Unique Insertion Sites Belong to Various Transposable Element Families

In the TIPs profile, 23,036 TE insertions were found to be only present in a single rice accession, which were called singletons. Subsequently, the low-quality and redundant singletons were removed through four strict filtering steps (Supplementary Figure 1; See section “Materials and Methods”). Finally, 10,924 high-confidence singletons were retained for further analysis (Supplementary Table 1). The hierarchical classification showed that more singletons resulted from active transposition of retrotransposons than from DNA transposons (6,735 versus 4,189) (Table 1). All 11 TE superfamilies were identified in our dataset (Table 1), but the number of singletons varied substantially across different superfamilies, implying distinct current activity. The loci of *Copia* were approximately twice more than *Gypsy* (4,307 versus 2,390; Table 1) despite that both belong to LTR type. For TIR elements, *PIF/Harbinger* exhibited the highest singletons number (2,015), while *EnSpm/CACTA*, by contrast, had the lowest (291 singletons).

**TABLE 1:** The classification of 10,924 singletons identified from 3,000 natural rice accessions.

Class	Type	Superfamily	No. of Singletons
Retrotransposon			6,735
	LTR		6,712
		*Copia*	4,307
		*Gypsy*	2,390
		*TRIM*	15
	LINE		16
		*L1*	16
	SINE		7
		*tRNA*	7
DNA transposon			4,189
	TIR		4,138
		*PIF/Harbinger*	2,015
		*Tc1/Mariner*	932
		*Mutator*	525
		*hAT*	375
		*EnSpm/CACTA*	291
	Helitron		51
		*Helitron*	51

**FIGURE 1:**
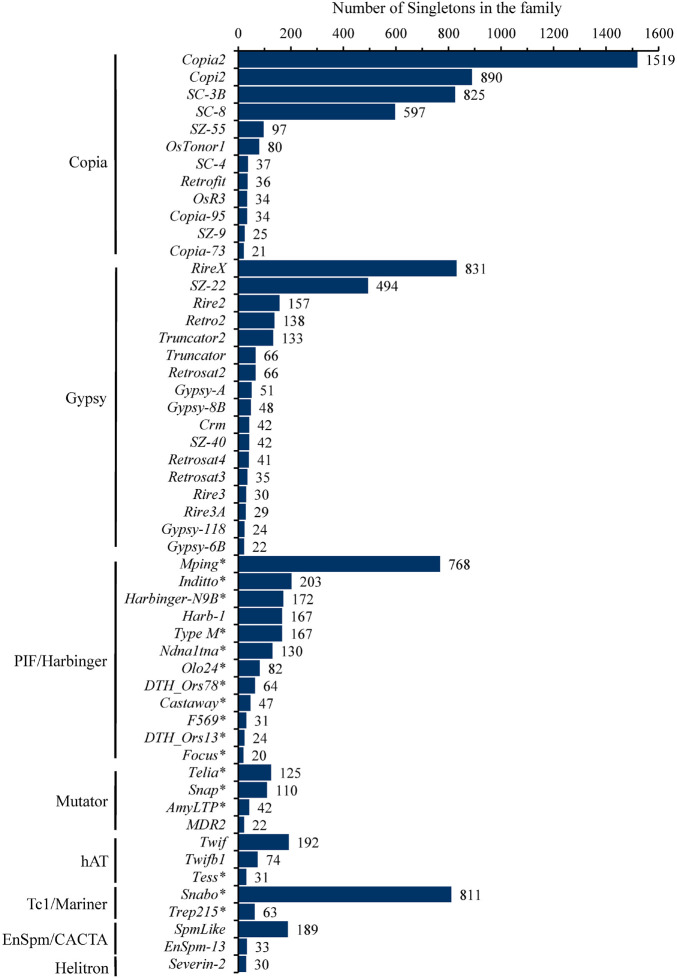
The number of singletons for each TE family. The 52 TE family belong to *Copia*, *Gypsy*, *PIF/Harbinger*, *Mutator*, *hAT*, *Tc1/Mariner*, *En/Spm/CACTA*, *Helitron* having more than 20 singletons were shown. Among them, eight families (*Copia2*, *Copi2*, *SC_3B*, *SC_8* from *Copia*; *RireX*, *SZ_22* from *Gypsy*; *mPing* from *PIF/Harbinger*; *Snabo* from *Tc1/Mariner*) harbored at least about five hundred singletons, far more than others, suggesting that they possessed the highest level of current activity in 3,000 natural rice accessions. The asterisk appended to the name of TEs denoted that the corresponding family was also annotated as MITEs.

These singletons were further classified into 251 TE families, with the number of singletons in each family ranging from 1 to 1,519 (Supplementary Table 2). In particular, eight families, including four *Copia* (*Copia2*, *Copi2*, *SC-3B*, *SC-8*), two *Gypsy* (*RireX*, *SZ-22*), one *PIF*/*Harbinger* (*mPing*) and one *Tc1*/*Mariner* (*Snabo*), had the maximum singletons and accounted for 61.7% (6,735/10,924) of the total singletons (Figure 1 and Supplementary Table 2), suggesting that they probably possess the highest current activity in 3,000 natural rice accessions. In addition, 53 of 251 families possessed at least 20 singletons and together contributed 91.9% (10,041/10,924) of the total singletons (Figure 1 and Supplementary Table 2). Of them, 12, 17, and 12 were from *Copia*, *Gypsy* and *Harbinger* superfamilies, respectively, while *Mutator*, *hAT*, *Mariner*, *CACTA*, *Helitron* superfamilies had only 1 to 4 families (Figure 1). Notably, 17 of 24 DNA transposon families belonged to MITEs, and accounted for 80.3% (2,890/3,597) of DNA transposons, indicating that most of the active DNA transposons were short non-autonomous elements in rice genome.

### Integration Preference of Transposable Elements Revealed by Singletons

**FIGURE 2:**
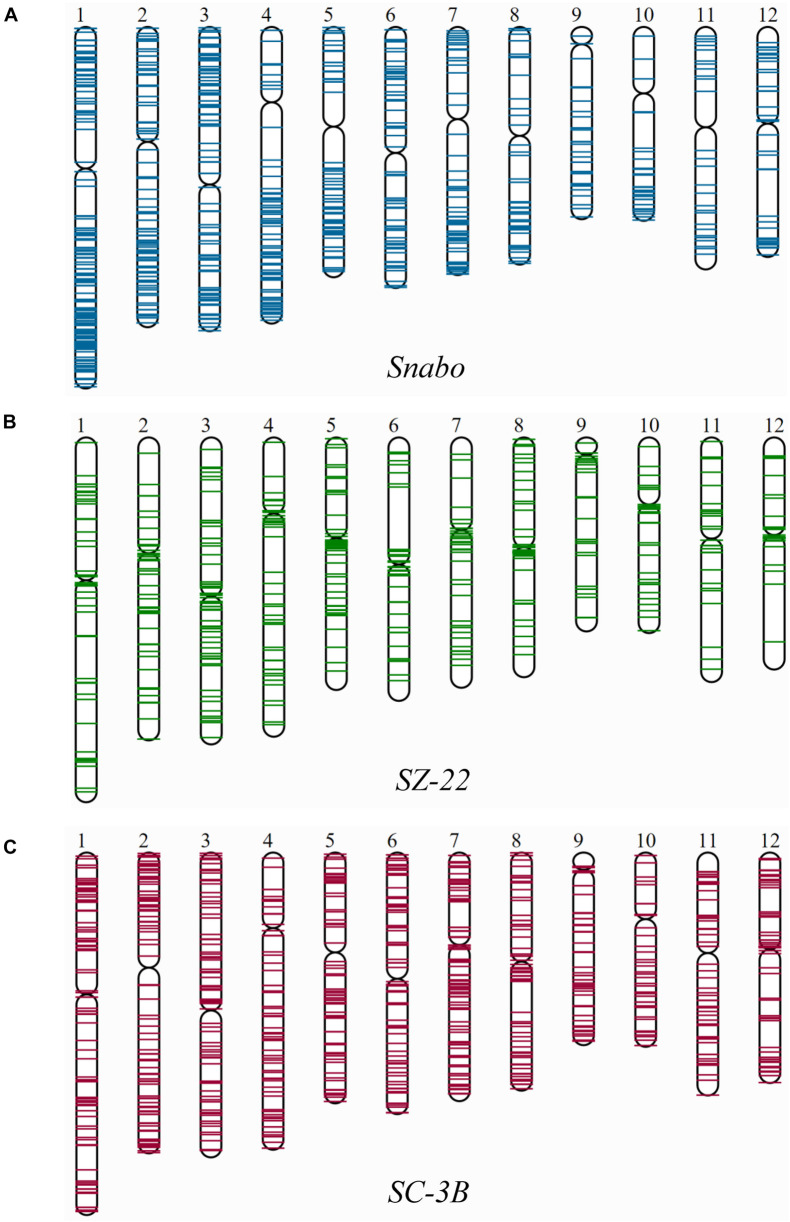
The distribution of singletons from individual TE family on 12 rice chromosomes. Only three representative TE families were shown, which exhibited distinct distribution patterns on rice chromosomes. **(A)** The *Snabo* family was observed to be inclined to integrate into the chromosomal arm region compared to the pericentromeric region by mapping the 811 singletons to 12 rice chromosomes; **(B)** the *SZ-22* family was observed to be biased toward the pericentromeric regions by mapping the 494 singletons in the family; **(C)** the *SC-3B* family showed no obvious insertion preference between the two chromosomal backgrounds.

Since the singleton was newly inserted into the genome, it was almost impossible to be affected by the selection or chromosome recombination. Therefore, the distribution of singletons on chromosomes could truly reflect the transposition characteristics. Fifty-three TE families harboring at least 20 singletons were then mapped on 12 rice chromosomes, and divided into three categories: pericentromere preference, chromosomal arm preference and unbiased distribution (Figure 2 and Supplementary Table 3). There were 38 (71.7%) TE families were significantly inclined to the chromosomal arm region, which included 79.3% (7,960/10,041) singletons [71.8% (4,630/6,444) retrotransposons and 92.6% (3,330/3,597) DNA transposons]. For instance, the *Snabo* family, whose average density in the chromosomal arm region was 6.4 times higher than that in the pericentromere region (Figure 2A; *p*-value < 7.31E-07, paired *t*-test). By comparison, only four families were biased toward the pericentromere region (Supplementary Table 3). A typical case was the *SZ-22* family, whose average density in pericentromere region was 5.6 times higher than that in the chromosomal arm region (Figure 2B; *p*-value = 1.60E-03, paired *t*-test). Interestingly, all the four families belonged to the *Gypsy* superfamily, accounting for 30.7% (691/2,249) of the Gypsy singletons. The remaining 11 families had no significant insertion preference between the two chromosomal backgrounds (Supplementary Table 3), although they might be unevenly distributed along the chromosomes, such as *SC-3B* (Figure 2C; *p*-value = 0.1291, paired *t*-test).

When integrating into new positions, TEs typically result in the target sites duplication (TSD), which represent the hotspots of TE insertions. To explore the mechanism underlying the biased distribution, TSDs of 5,849 singletons were extracted from both forward and reverse directions flanking the insertion sites (Supplementary Figure 2). The results showed that TSDs of 12 families in *Harbinger* superfamily had the conserved sequences of TAA/TTA (Supplementary Table 4 and Supplementary Figure 3). The *Telia* family in the Mutator superfamily inserted specifically into AT-rich sequences (TAAATTATA), while the *MuDR2* family preferentially targeted CG-rich sequences (Supplementary Table 4 and Supplementary Figure 3). The remaining TE families almost had no conserved sequences in TSDs. Nevertheless, the TSD length of TE families in the same superfamily showed exact consistency. For example, the *Copia* and *Gypsy* superfamilies had 5 bp TSDs, while *hAT* and *CACTA* superfamilies had 8 and 3 bp TSDs, respectively (Supplementary Table 4 and Supplementary Figure 3).

In addition, we analyzed the association of singletons with the annotated genes to understand whether they potentially affect the function of genes. Of the 10,924 singletons, 926 (8.5%) and 1,993 (18.2%) were inserted into the coding regions and introns of genes (Supplementary Table 5), respectively, which may disrupt gene function through insertion mutagenesis or altering splicing patterns. The 238 (2.2%) and 409 (3.7%) singletons were located in the 5′-UTR and 3′-UTR of the genes, respectively (Supplementary Table 5). In addition, 479 (4.4%) and 277 (2.5%) loci were found within the 200 bp upstream and downstream sequences adjacent to the annotated genes (Supplementary Table 5), respectively, which were likely to change gene expression by providing putative *cis*-regulatory elements or epigenetic regulation.

### Extensively Current Activity of Transposable Elements and Potentially Highly Active Transposable Elements in Rice

**FIGURE 3:**
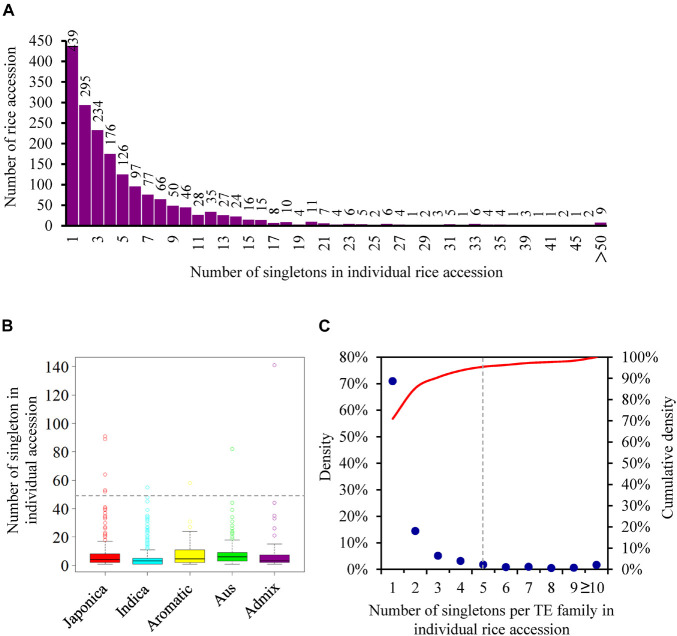
The distribution of singletons among different rice accessions. **(A)** The distribution of the number of singletons in individual rice accession; **(B)** the distribution of the number of singletons in rice accession from five distinct groups. Each boxplot shows the distribution of the number of singletons owned by each rice accession in the corresponding group. The median represented the middle number of singletons among all rice accession from the same group. The number corresponding to the gray dashed line is 50; **(C)** the distribution of the number of singletons per TE family in individual rice accession. The red curve represented the cumulative density.

The 10,924 singletons were distributed in 1,864 of 3,000 rice accessions (Supplementary Table 6), with an average of 5.86 insertions per accession, reflecting the extensive activity of TEs in 3,000 natural rice accessions. Among different accessions, the number of singletons displayed large differences, varying from 1 to 141 (Figure 3A and Supplementary Table 6). Particularly, nine rice accessions, including five japonicas (IRIS_313-15904; IRIS_313-7885; IRIS_313-10228; IRIS_313-10703; IRIS_313-10562) and one each of indica (B227), aus/boro (IRIS_313-11413), aromatic (IRIS_313-11217), admix (IRIS_313-9445), contributed more than 50 singletons (Figure 3A and Supplementary Table 6), suggesting that they may carry more active TEs. When categorizing rice accessions according to five distinct groups (japonica, indica, aus, aromatic, and admix), it was observed that the indica accessions had the least number of singletons on average (4.75 singletons per accession), followed by japonica (6.58 singletons per accession), while aus, aromatic and admix accessions possessed relatively more singletons, with an average of 8.01, 8.43, and 8.71 insertions per accession, respectively (Figure 3B and Supplementary Table 6).

**FIGURE 4:**
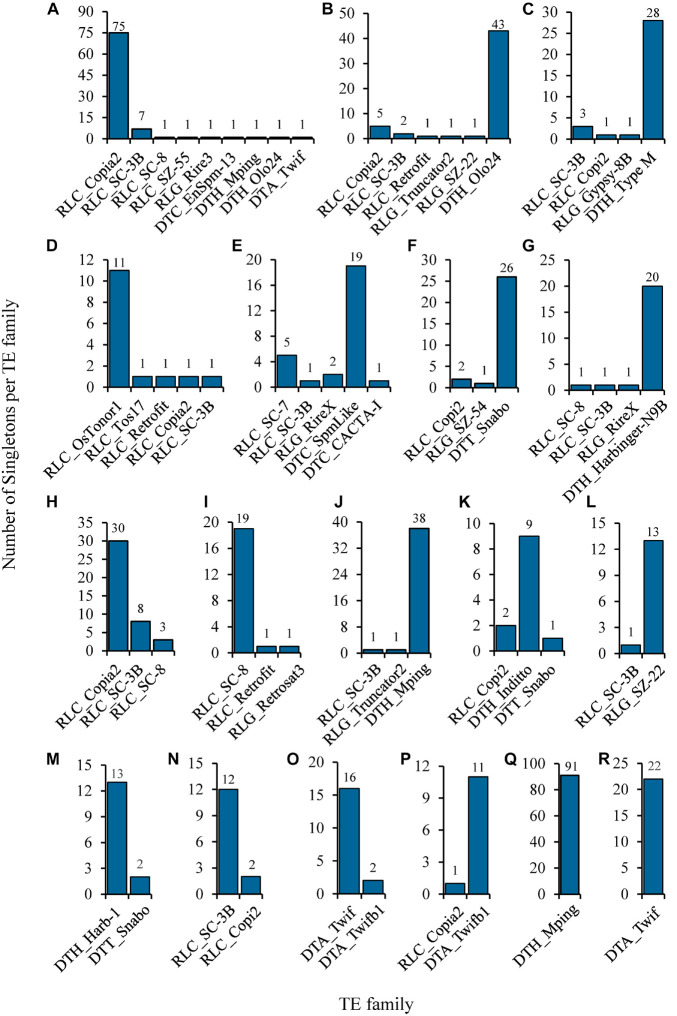
Selected examples of potentially highly-active TEs identified from natural rice accessions. The histograms show the composition of singletons identified in each of the following rice accessions: **(A)** Trop/IRIS_313-7885; **(B)** Trop/IRIS_313-10703; **(C)** indx/IRIS_313-12300; **(D)** Subtrop/IRIS_313-11897; **(E)** aus/IRIS_313-11482; **(F)** Ind2/IRIS_313-9740; **(G)** aus1/IRIS_313-10930; **(H)** Trop/IRIS_313-10758; **(I)** Aro/IRIS_313-11068; **(J)** Temp/IRIS_313-11655; **(K)** ind3/IRIS_313-12142; **(L)** Subtrop/IRIS_313-8580; **(M)** ind2/IRIS_313-8703; **(N)** Indx/B090; **(O)** Temp/IRIS_313-11800; **(P)** Trop/IRIS_313-9550; **(Q)** Temp/IRIS_313-15904; **(R)** Temp/IRIS_313-8149. The three-letter prefix in the TE family name represents the superfamily to which it belongs. Its meaning is as follows: RLC: Copia; RLG: Gypsy; DTC: EnSpm/CACTA; DTH: PIF/Harbinger; DTA: hAT; DTT: Tc1/Mariner.

The identified singletons may be from different TE families in the individual rice accession. Therefore, they were further classified by families in each accession (Supplementary Table 7). Most TE families possessed only 1 to 4 singletons in all accessions (Figure 3C and Supplementary Table 7), while 35 families harbored at least 5 singletons in one or more rice accessions (Figure 4 and Supplementary Table 8), suggesting that they probably have highly current activity in the corresponding accession. For instance, *Copia2* family contributed 75 of 89 identified singletons in the tropical japonica accession IRIS_313-7885 (Figure 4A and Supplementary Table 8); *mPing* family created up to 91 singletons in the temperate japonica accession IRIS_313-15904, which was the only active family detected in this accession (Figure 4Q and Supplementary Table 8); *Olo24* contributed 43 of 53 identified singletons in the tropical japonica accession IRIS_313-10703 (Figure 4B and Supplementary Table 8). Of 35 TE families, 20 (11 retrotransposon and 9 DNA transposon families) displayed potentially high activity in multiple (≥3) rice accessions (Supplementary Table 8). Particularly, *Copia2* family maintained potentially high activity in up to 92 rice accessions, with the number of singletons per accession varying from 5 to 75.

### Variable Activity of Transposable Elements Among Different Rice Groups

**FIGURE 5:**
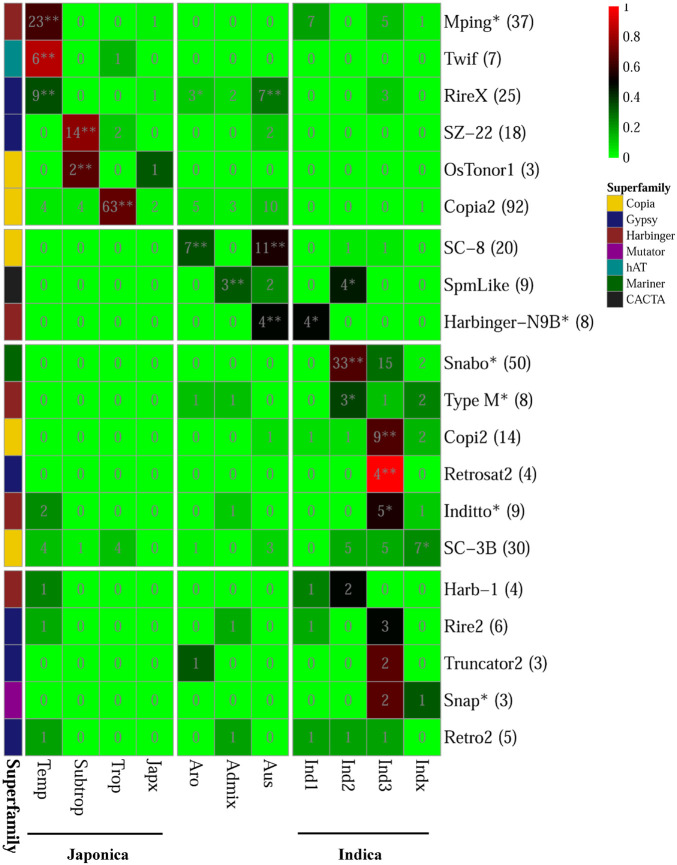
Distribution of rice accessions carrying PHA TE among different variety groups/subgroups. The numbers in parentheses represented the total number of rice accessions carrying the corresponding highly active TE in 3,000 natural rice accessions. The asterisk appended to the name of TE family denoted that the corresponding family was also annotated as MITE. The background color displayed in the cell represented the ratio of the number of rice accessions carrying the corresponding highly active TE in the variety group to total number of rice accessions carrying the corresponding highly active TE in 3,000 natural rice accessions. In addition, the asterisk and double asterisks in the cells represented significant and extremely significant level in the Fisher’s exact test for the enrichment analysis, respectively.

For the 20 PHA TE families, we analyzed the distribution of their PHA accessions (referring to the rice accessions carrying the corresponding PHA TEs) among different groups and subgroups, where both indica and japonica were further subdivided into four subgroups (indica: indica1, indica2, indica3, indicaX; japonica: temperate japonica, tropical japonica, subtropical japonica, japonicaX) (Figure 5 and Supplementary Table 9). As a result, six TE families were significantly enriched in the japonica group (*p*-value < 0.05, Fisher’s exact test), including *mPing*, *Twif* and *RIREX* in the temperate japonica subgroup, with 62.2% (23/37), 85.7% (6/7) and 36.0% (9/25) of PHA accessions being in this subgroup, respectively; *SZ-22* and *OsTonor1* in the subtropical japonica subgroup, with 77.8% (14/18) and 66.7% (2/3) of PHA accessions being subtropical japonica, respectively; *Copia2* in the tropical japonica subgroup, with 68.5% (63/92) of PHA accessions being tropical japonica (Figure 5 and Supplementary Table 9). Similarly, eight TE families showed significant enrichment in the indica group (*p*-value < 0.05, Fisher’s exact test), including one (*Harbinger-N9B*) in the indica1 subgroup, three (*SpmLike*, *Snabo* and *Type M*) in the indica2 subgroup, three (*Copi2*, *Retrosat2*, *Inditto*) in the indica3 subgroup and one (*SC-3B*) in the indicaX subgroup. In addition, two (*RIREX* and *SC-8*), one (*SpmLike*) and three (*RIREX*, *SC-8* and *Harbinger-N9B*) TE families presented significant enrichment in the aromatic, admix and aus group, respectively (*p*-value < 0.05, Fisher’s exact test; Figure 5 and Supplementary Table 9). For the remaining five TE families (*Harb-1*, *Rire2*, *Truncator2*, *Snap*, and *Retro2*), no significant enrichment could be detected in any group or subgroup.

To understand the evolutionary relationship among these PHA rice accessions, they were mapped to the phylogenetic tree of 3,000 rice accessions constructed from SNPs in the previous study (Mansueto et al., 2017). Most of the PHA accessions corresponding to the same TE family clearly gathered together to form one or several clusters, rather than scattered randomly throughout the tree (Supplementary Figure 4), suggesting that they may share a common origin of activity.

## Discussion

### The Large-Scale Survey of Active Transposable Elements at the Population Scale

There was an eternal competition between TEs and hosts, which caused the birth and extinction of transposition activity to occur repeatedly as the genome evolved. It was important to determine which TEs were still active in the genome. Previous studies identified a few of active TEs from rice, but they only focused on a single TE family in one or few rice varieties (Hirochika et al., 1996; Jiang et al., 2003; Picault et al., 2009; Dong et al., 2012; Cheng et al., 2015; Tang et al., 2019). Considering the difference in transposition activity among different varieties (Hirochika et al., 1996; Jiang et al., 2003; Naito et al., 2006), it prevented us from comprehensively understanding the current activity of rice TEs. In this study, we systematically surveyed the activity of all TEs in 3,000 natural rice accessions by detecting UIS based on a computational approach. 10,924 high-confidence singletons from 251 TE families were identified from 1,864 of 3,000 rice accessions. It should be the first investigation for any organism to detect potential active TEs at the population scale.

### Active Mobilization of Transposable Elements Under Normal Growth Conditions Rather Than Artificial Induction

The known active TEs identified from rice were mostly induced by artificial treatment up to now. *Tos17* (Hirochika et al., 1996), *mPing* (Jiang et al., 2003), *Lullaby* (Picault et al., 2009), *mGing* (Dong et al., 2012), *Karma* (Komatsu et al., 2003), *nDaiZ* (Huang et al., 2009) were activated under tissue culture; *dTok* (Moon et al., 2006), *nDart* (Tsugane et al., 2006), *mJing* (Tang et al., 2019), *Osr7*, *Osr23* (Wang et al., 2009) were isolated from the progeny of intraspecific or interspecific hybridization. By comparison, their active mobilization was rarely found under normal growth conditions. One exception was *mPing*, which possessed very high activity in the temperate japonica cultivar Gimbozu EG4 and three related landraces (Aikoku A157, Aikoku A119, Aikoku A123) (Naito et al., 2006). These rice cultivars carrying naturally active *mPing* played a crucial role in biology research on TEs. They were recruited to successfully address two important questions concerning TE bursts, namely how TE achieved very high copy number without killing their host (Naito et al., 2006, 2009), and how the burst was sustained for generations without triggering host’s silencing mechanisms (Lu et al., 2017).

The active TEs obtained in this study were identified from rice cultivated population. Therefore, their active transposition occurred under normal growth conditions. Compared with that derived from artificial induction, these active TEs could truly reflect the active status of TEs in the current rice genome. These naturally active TEs were likely to be activated by exogenous stresses. Previous studies have shown that TEs were capable of responding to various biotic and abiotic stresses (Grandbastien, 1998), such as wounding (Mhiri et al., 1997), radiation (Questa et al., 2010), cold (Ivashuta et al., 2002; Naito et al., 2009; Butelli et al., 2012), heat (Ito et al., 2011), salt (Naito et al., 2009), as well as pathogen infection (Grandbastien et al., 2005; Buchmann et al., 2009). The 3,000 diverse rice accessions involved in this study were grown worldwide and faced to different ecological and geographical environments. They were challenged by a variety of biotic and abiotic stresses during domestication, which provided abundant external conditions for TEs activation. The active transposition of these TEs enabled the host to rapidly acquire genetic diversity to cope with altering environments.

Another possibility was that the activation of these TEs was attributed to mutations in genes involved in the epigenetic pathway. Under normal conditions, the activity of most TEs was strictly repressed by epigenetic regulation. However, their active transposition could be triggered when the related genes were mutated during rice cultivation. For instance, disruption of rice SDG714, encoding a histone H3K9-specific methyltransferase, reduced the levels of CG and CHG methylation as well as H3K9 methylation and promoted frequent transposition of *Tos17* (Ding et al., 2007). The loss function of JMJ703 increased H3K4me3 and enhanced the transposition activity of *Karma* element (Cui et al., 2013). In this study, some rice accessions possessed several or even dozens of active TE families simultaneously (Supplementary Table 7), suggesting that their activities may be resulted from the relaxation of the epigenetic silencing.

The activation of TEs may also be due to alteration of TE sequences. It was reported that the burst of *mPing* in the domesticated rice accessions was associated with the acquisition of two variants of the transposase loci, *Ping16A* and *Ping16A_Stow* (Chen et al., 2019). Meanwhile, these naturally active TEs would be ideal candidates for elucidating the molecular mechanisms underlying the activation of TEs, and investigating the interaction between TEs and the host genome. In addition, they would also be potentially valuable resources for tagging system of gene discovery in rice and other important corps.

### A High-Throughput Strategy for Identifying Active Transposable Elements

Since active TEs are rare, it is very challenging to recognize them from numerous inactive elements. At present, the active TEs were mainly identified by the following methods. Firstly, inspecting the sequences between different copies to discover identical copies. It is based on the fact that new insertions arising from the current activity don’t accumulate mutations. The active *mPing* was initially discovered by this method (Jiang et al., 2003). However, it is only applicable to the assembled genomes, since this method require full-length sequences of the TE. Secondly, characterizing spontaneous mutants. Most of the known active TEs in rice were isolated by this method up to now (Moon et al., 2006; Tsugane et al., 2006; Huang et al., 2009; Wang et al., 2009; Tang et al., 2019). It is undeniable that more active TEs will be revealed as more mutants are analyzed in the future. However, the acquisition of active TEs by this method is largely accidental, since the mutants caused by active transposition account for relatively small proportion. Thirdly, detecting transcriptional activity (Picault et al., 2009). Regardless of the retrotransposon or DNA transposon, the expression of genes encoding the enzymes required for transposition is a necessary prerequisite for successful transposition. Therefore, we can preliminarily speculate whether TEs are still active by detecting their transcripts or not. Nevertheless, the active transcription of TEs don’t ensure that they are ultimately successfully transposed, because the hosts have evolved multiple mechanisms to regulate transposition activity in the post-transcription stages (Slotkin and Martienssen, 2007).

The emergence of new insertions in the genome was the most powerful and direct evidence to prove the active transposition. These loci were expected to be only present in one accession of the whole population, as their transposition occurred after divergence between different accessions. Therefore, it was a feasible strategy to identify active TEs by detecting singleton insertions from a large population. However, it was almost impossible to perform such research by traditional experimental methods because of the enormous workload. Recently, the development of next-generation sequencing technology has provided an excellent opportunity for efficient researches on genetic variation. Here we provided a high-throughput strategy for capturing active TEs from the resequenced population. The main content of this method was to construct a comprehensive profile of TIPs by analyzing the resequenced data from the population using our previously developed pipeline (Liu et al., 2020b). It consisted of the identification of TE loci and the genotyping of each locus in the population. Then, singleton insertions were screened out from the profile. The advantage of this method was that it was applicable to all types of TEs and was capable of detecting a large number of active TEs from the population in a single analysis. It could also be applied directly to other species when the whole-genome resequenced data for their population were available.

## Data Availability Statement

Publicly available datasets were analyzed in this study. This data can be found here: http://ibi.zju.edu.cn/Rtrip/index.html.

## Author Contributions

J-HX and ZL contributed to the conception and design of the study and wrote the manuscript. ZL, HZ, YY, M-XW, Y-CZ, E-KY, MA, KS, M-HD, and J-HX analyzed the data. HZ and Y-CZ provided the computer resource. All authors read and contributed to the manuscript.

## Conflict of Interest

M-HD was employed by Zhejiang Zhengjingyuan Pharmacy Chain Co., Ltd., and Hangzhou Zhengcaiyuan Pharmaceutical Co., Ltd., Hangzhou, China. The remaining authors declare that the research was conducted in the absence of any commercial or financial relationships that could be construed as a potential conflict of interest.

## Publisher’s Note

All claims expressed in this article are solely those of the authors and do not necessarily represent those of their affiliated organizations, or those of the publisher, the editors and the reviewers. Any product that may be evaluated in this article, or claim that may be made by its manufacturer, is not guaranteed or endorsed by the publisher.

## Footnotes

1
http://ibi.zju.edu.cn/Rtrip/method.html

2
https://github.com/lanceliu2018/FrontPlantSci_OsaTE

3
http://rice.plantbiology.msu.edu/

## Supplementary Material

Supplementary Figure 1 The outline of the procedure for the identification of high-confidence singleton insertions. In the TIPs profile, 27,531 of 60,743 TE loci were detected simultaneously from both forward and reverse directions flanking the TE insertion. Therefore, each of them has two records corresponding to the forward and reverse directions in the profile. In total, there are 88,274 records corresponding to 60,743 TE loci in the profile. We used these data as the starting point for screening singleton insertions. As a result, 23,036 of 88,274 insertions were found to be singletons. Among them, 2,924 singletons were heterozygous, 2,343 singletons had missing calls in more than 10% of rice accessions, 972 singletons had no sufficient supporting reads, 5,873 singletons had two records corresponding to the forward and reverse directions. Finally, a filtered dataset consisting of 10,924 high-confidence non-redundant TE singletons was obtained.

Supplementary Figure 2 The general schematic view of the method to detect TSDs for singleton loci that were identified from both forward and reverse directions around the insertion sites.

Supplementary Figure 3 Analysis of sequence conservation of TSD for different TE families. The sequence logos were generated by WebLogo3 (Schneider and Stephens, 1990; Crooks et al., 2004).

Supplementary Figure 4 Distribution of rice accessions carrying PHA TE in the phylogenetic tree of 3,000 natural rice accessions, which was constructed from SNPs in the previous study (Mansueto et al., 2017).

Supplementary Table 1 The information of singletons identified from 3,000 natural rice accessions.

Supplementary Table 2 The number of singletons in the 251 TE families.

Supplementary Table 3 The density of singletons by each family on the 12 chromosomes of rice.

Supplementary Table 4 TSD of 5,849 singletons detected from both ends of the insertions.

Supplementary Table 5 The information of the singletons associated with genes.

Supplementary Table 6 The number of singletons in each rice accession and variety group.

Supplementary Table 7 The classification of singletons by TE family in each rice accession.

Supplementary Table 8 The PHA TEs identified from 3,000 natural rice accessions.

Supplementary Table 9 The distribution of rice accessions carrying PHA TEs among different variety groups and subgroups.
