# Supplementary material for: Extensively Current Activity of Transposable Elements in Natural Rice Accessions Revealed by Singleton Insertions
Source: Front Plant Sci. 2021 Sep 28;12:745526. doi: 10.3389/fpls.2021.745526 (PMC8505701; doi:10.3389/fpls.2021.745526)

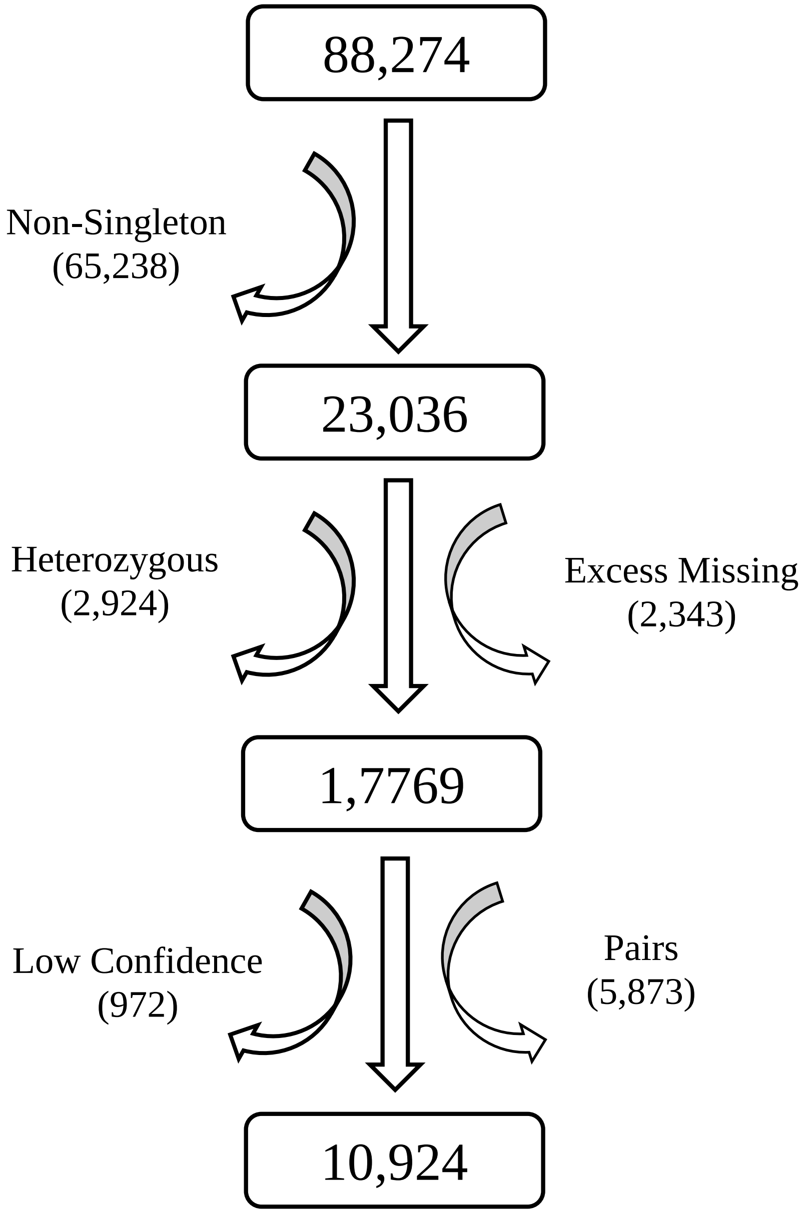
88,274
Non-Singleton
(65,238)
23,036
Heterozygous
(2,924)
Excess Missing
(2,343)
1,7769
Low Confidence
(972)
Pairs
(5,873)
10,924

Figure S1

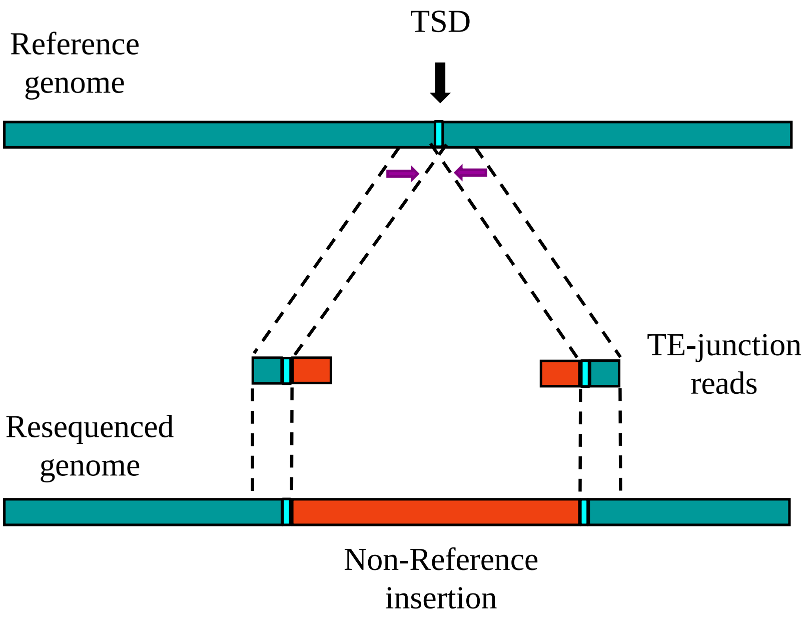
TSD
Reference genome
TE-junction reads
Resequenced genome
Non-Reference insertion

Figure S2

PIF/Harbinger

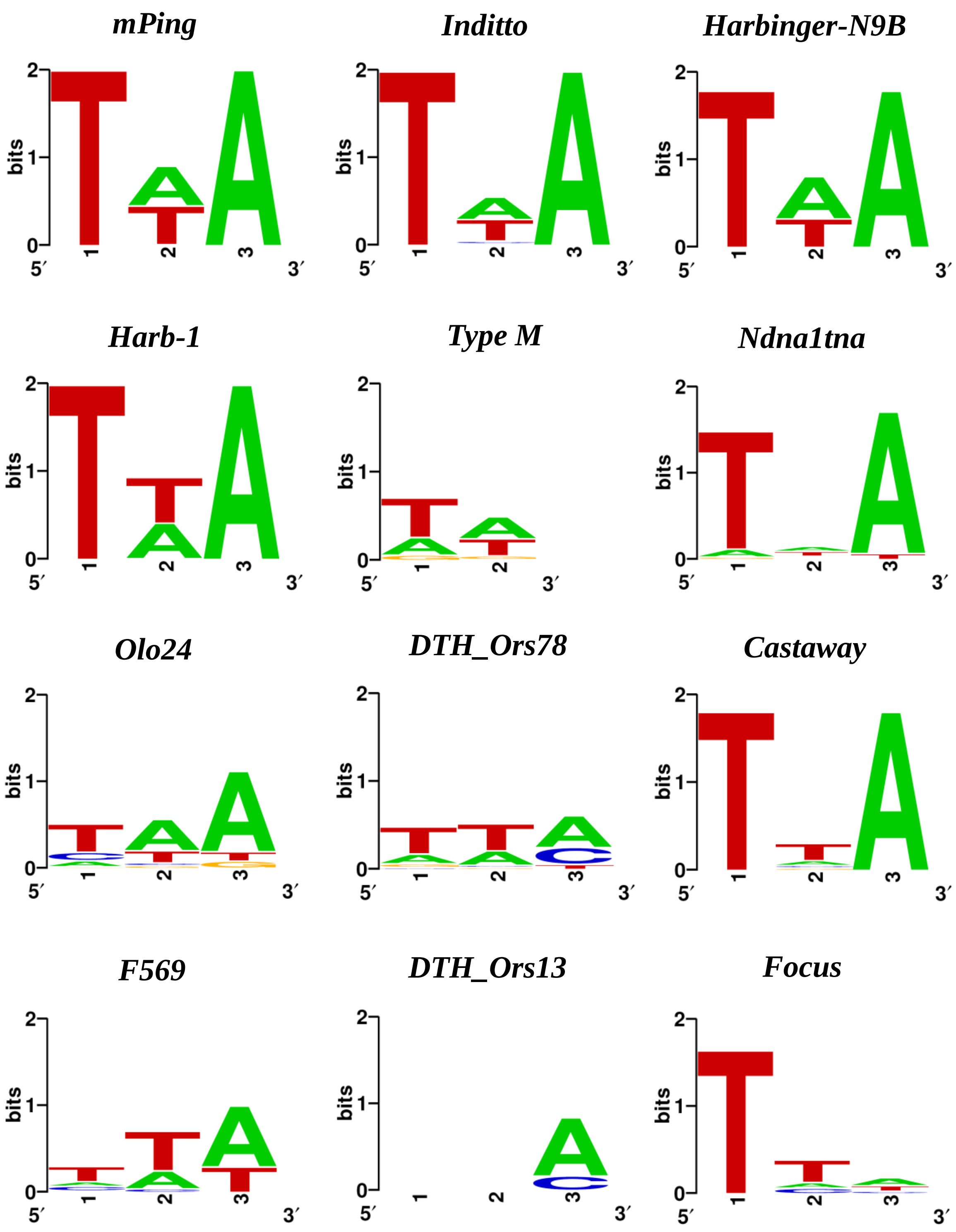

Mutator

Telia

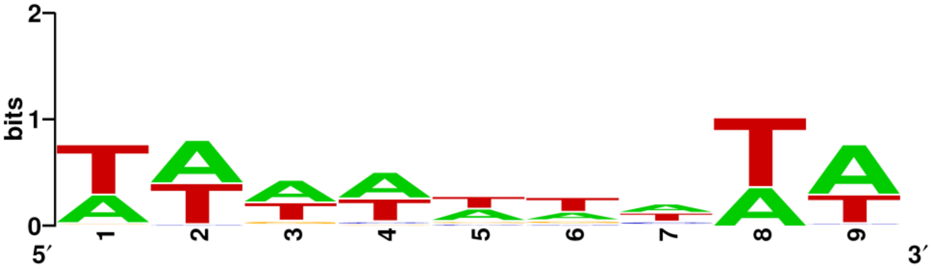

AmyLTP

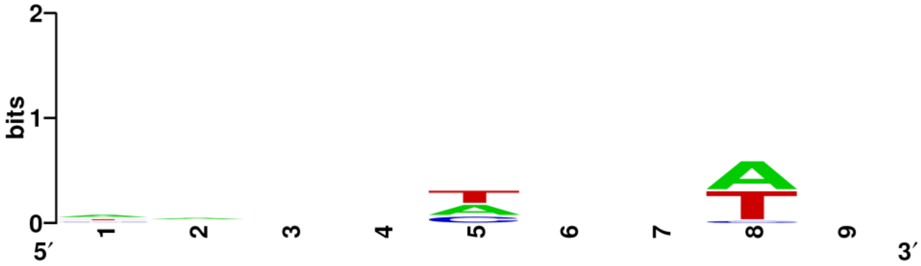

MuDR2

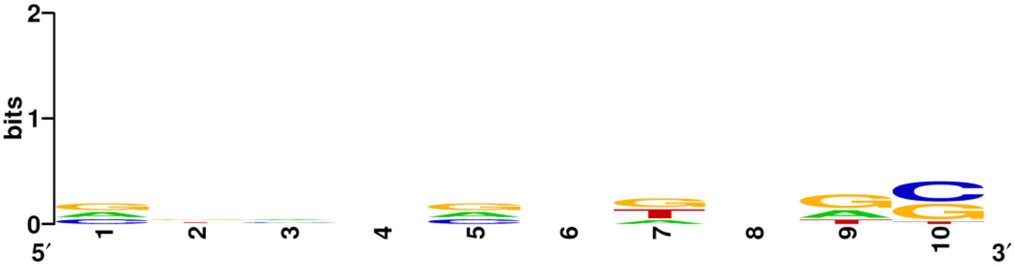

Copia

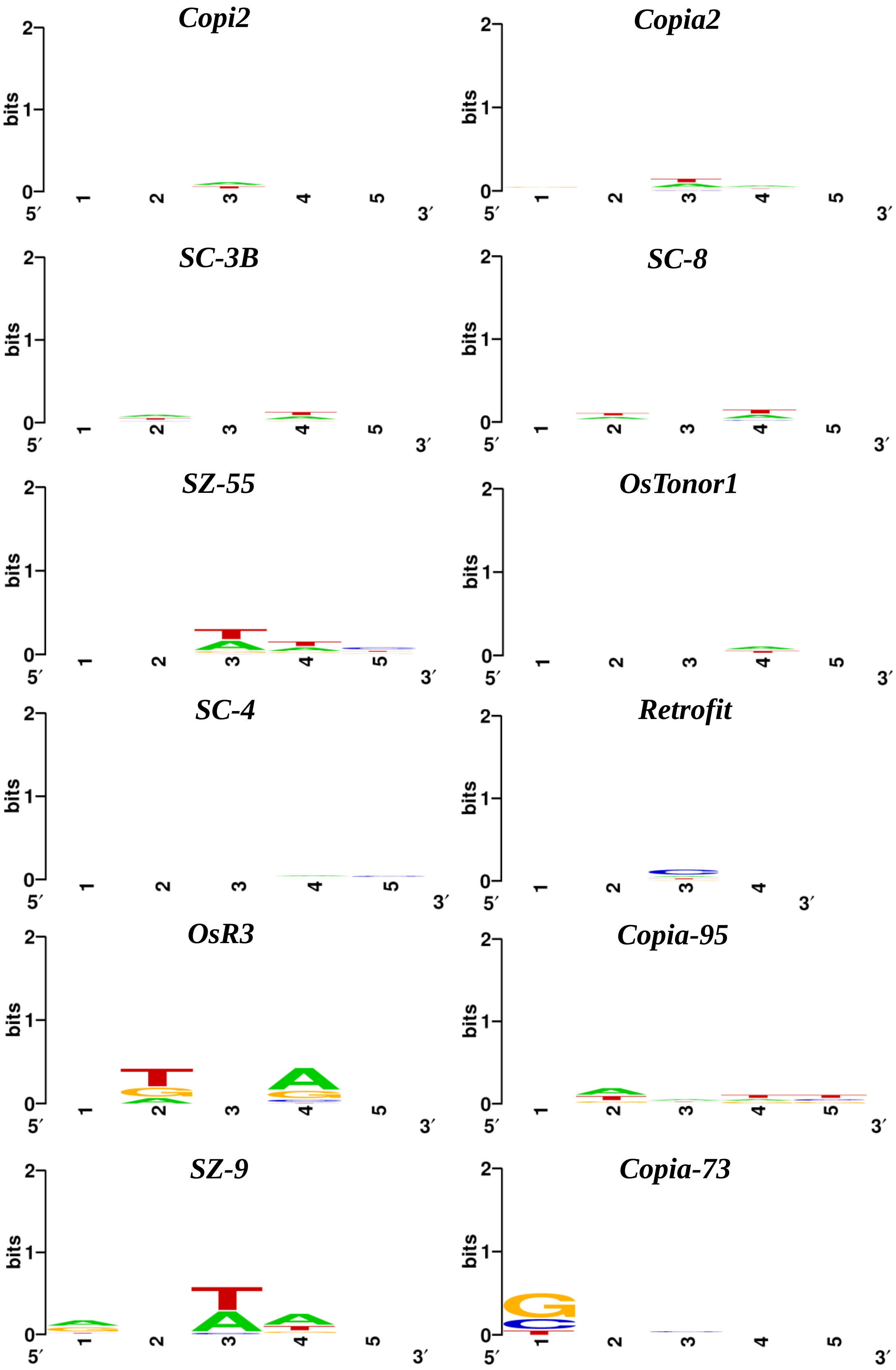
Copi2
Copia2
SC-3B
SC-8
SZ-55
OsTonor1
SC-4
Retrofit
OsR3
Copia-95
SZ-9
Copia-73
bits
0
1
2
5′
3′
1
2
3
4
5

Gypsy

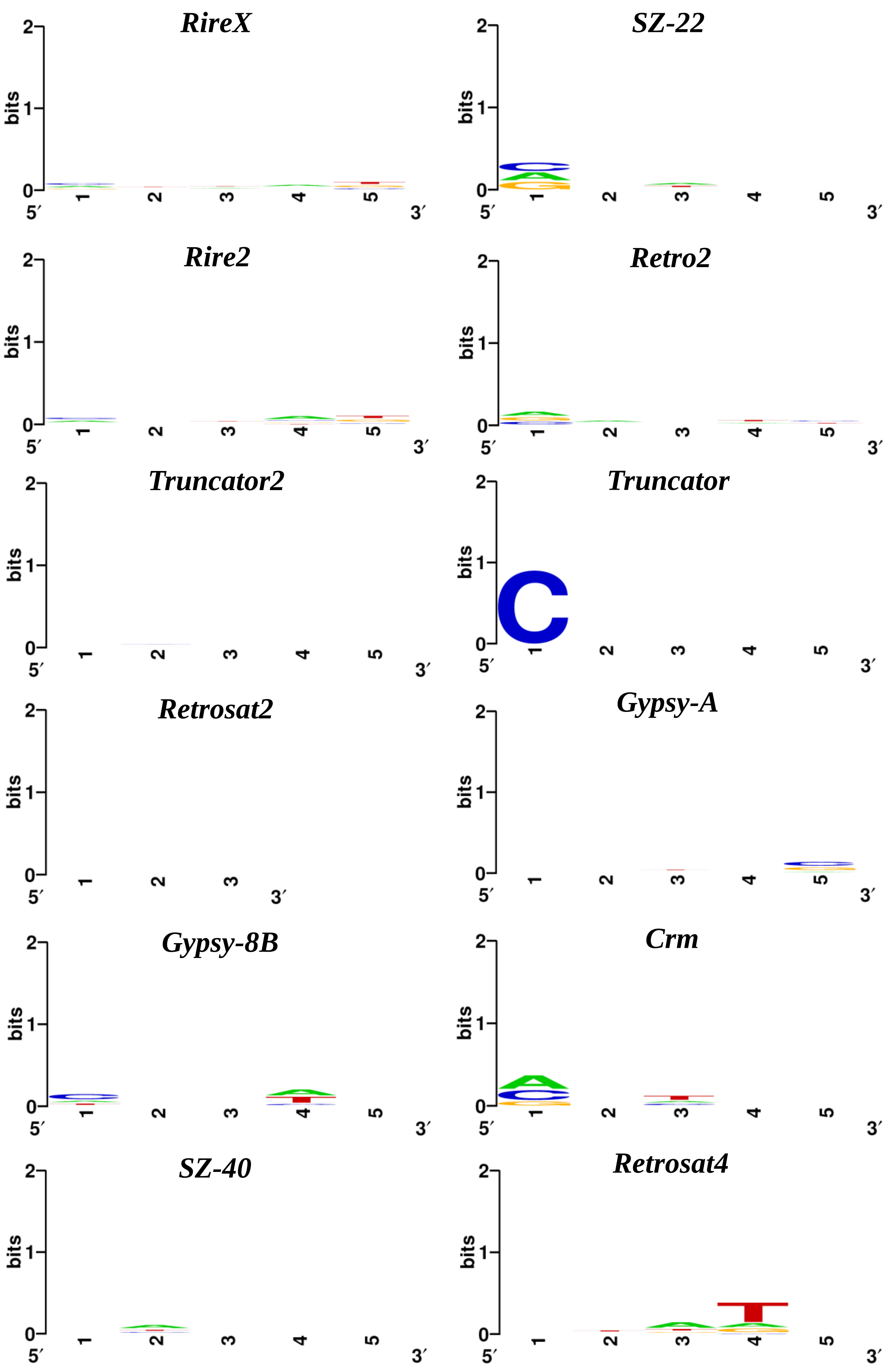
RireX
SZ-22
Rire2
Retro2
Truncator2
Truncator
Retrosat2
Gypsy-A
Gypsy-8B
Crm
SZ-40
Retrosat4
bits
5′
3′

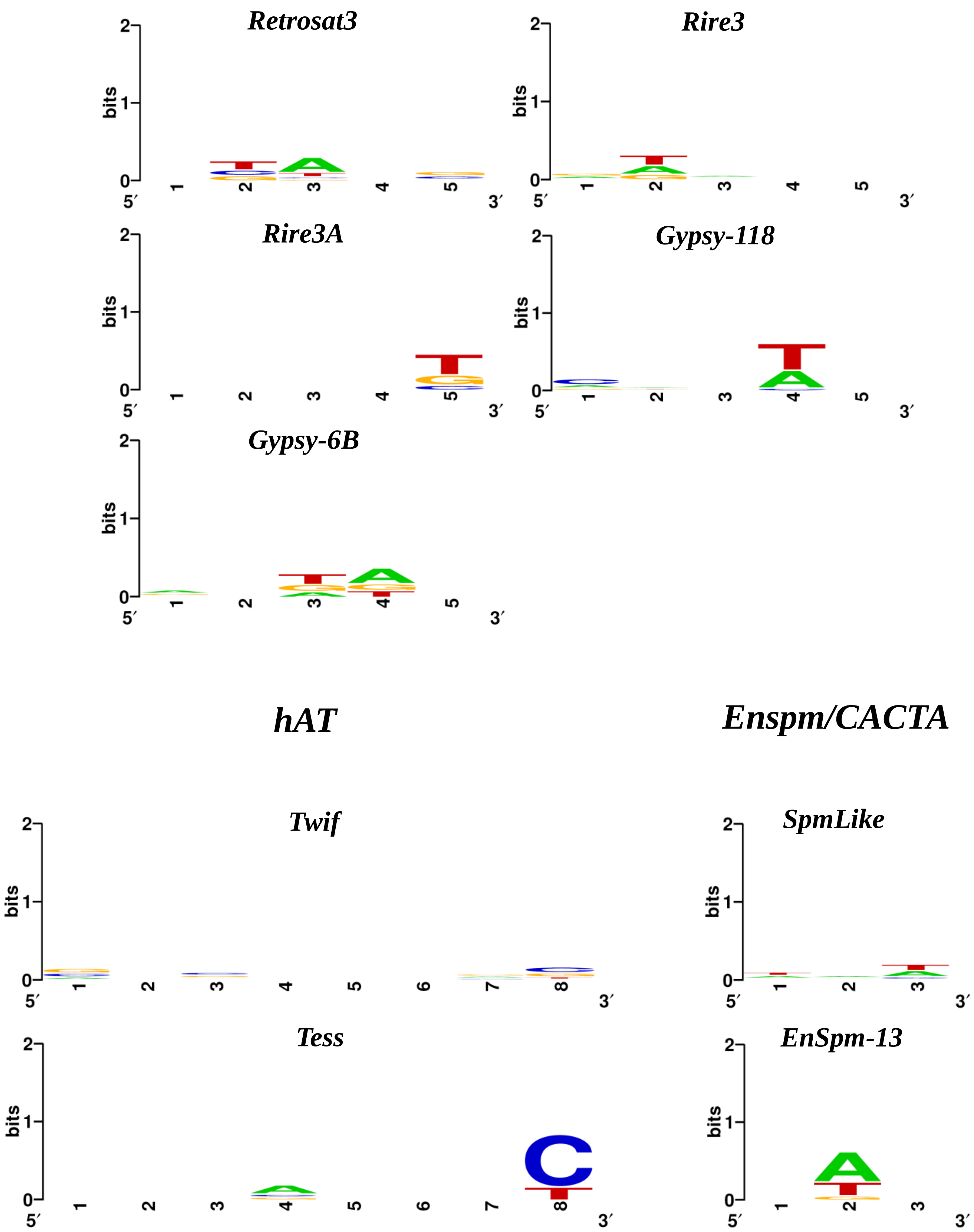
Retrosat3
Rire3
Rire3A
Gypsy-118
Gypsy-6B
hAT
Enspm/CACTA
Twif
SpmLike
Tess
EnSpm-13

Figure S3

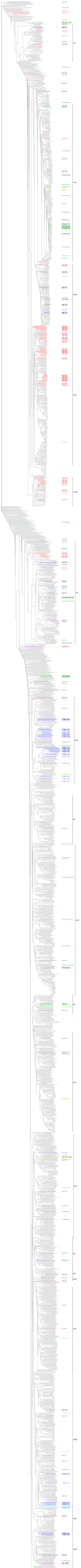

Supplement: Supplementary Figure 1 — The outline of the procedure for the identification of high-confidence singleton insertions. In the TIPs profile, 27,531 of 60,743 TE loci were detected simultaneously from both forward and reverse directions flanking the TE insertion. Therefore, each of them has two records corresponding to the forward and reverse directions in the profile. In total, there are 88,274 records corresponding to 60,743 TE loci in the profile. We used these data as the starting point for screening singleton insertions. As a result, 23,036 of 88,274 insertions were found to be singletons. Among them, 2,924 singletons were heterozygous, 2,343 singletons had missing calls in more than 10% of rice accessions, 972 singletons had no sufficient supporting reads, 5,873 singletons had two records corresponding to the forward and reverse directions. Finally, a filtered dataset consisting of 10,924 high-confidence non-redundant TE singletons was obtained. [file Data_Sheet_1.PDF]
